# Developing the intersectionality supplemented Consolidated Framework for Implementation Research (CFIR) and tools for intersectionality considerations

**DOI:** 10.1186/s12874-023-02083-4

**Published:** 2023-11-09

**Authors:** Isabel B. Rodrigues, Christine Fahim, Yasmin Garad, Justin Presseau, Alison M. Hoens, Jessica Braimoh, Diane Duncan, Lora Bruyn-Martin, Sharon E. Straus

**Affiliations:** 1 Department of Medicine, McMaster University, Hamilton, ON Canada; 2 Knowledge Translation Program, St. Michael’s Hospital, Unity Health Toronto, Toronto, ON Canada; 3 Clinical Epidemiology, Ottawa Hospital Research Institute, Ottawa, ON Canada; 4 School of Epidemiology and Public Health, University of Ottawa, Ottawa, ON Canada; 5 School of Psychology, University of Ottawa, Ottawa, ON Canada; 6 Department of Physical Therapy, University of British Columbia, Vancouver, BC Canada; 7 Department of Social Science, York University, Toronto, ON Canada; 8 Physician Learning Program, Cumming School of Medicine, University of Calgary, Calgary, AB Canada; 9 Research Institute for Aging, University of Waterloo, Waterloo, ON Canada; 10 Department of Medicine, University of Toronto, Toronto, ON Canada

**Keywords:** Intersectionality, Knowledge translation, Frameworks, Theories, Models, Implementation science, Equity, Diversity, Inclusion, Consolidated framework for implementation research, CFIR

## Abstract

**Background:**

The concept of intersectionality proposes that demographic and social constructs intersect with larger social structures of oppression and privilege to shape experiences. While intersectionality is a widely accepted concept in feminist and gender studies, there has been little attempt to use this lens in implementation science. We aimed to supplement the Consolidated Framework for Implementation Research (CFIR), a commonly used framework in implementation science, to support the incorporation of intersectionality in implementation science projects by (1) integrating an intersectional lens to the CFIR; and (2) developing a tool for researchers to be used alongside the updated framework.

**Methods:**

Using a nominal group technique, an interdisciplinary framework committee (n = 17) prioritized the CFIR as one of three implementation science models, theories, and frameworks to supplement with intersectionality considerations; the modification of the other two frameworks are described in other papers. The CFIR subgroup (n = 7) reviewed the five domains and 26 constructs in the CFIR and prioritized domains and constructs for supplementation with intersectional considerations. The subgroup then iteratively developed recommendations and prompts for incorporating an intersectional approach within the prioritized domains and constructs. We developed recommendations and prompts to help researchers consider how personal identities and power structures may affect the facilitators and inhibitors of behavior change and the implementation of subsequent interventions.

**Results:**

We achieved consensus on how to apply an intersectional lens to CFIR after six rounds of meetings. The final intersectionality supplemented CFIR includes the five original domains, and 28 constructs; the *outer systems and structures* and the *outer cultures* constructs were added to the outer setting domain. Intersectionality prompts were added to 13 of the 28 constructs.

**Conclusion:**

Through an expert-consensus approach, we modified the CFIR to include intersectionality considerations and developed a tool with prompts to help implementation users apply an intersectional lens using the updated framework.

**Supplementary Information:**

The online version contains supplementary material available at 10.1186/s12874-023-02083-4.

## Background

Health inequities are the unjust differences that certain groups encounter when attempting to access and receive optimal healthcare [1]. A few examples of populations disproportionally impacted by healthcare inequities include racialized individuals, Indigenous groups, socioeconomically underprivileged communities, and gender minorities [2, 3]. Even when access-related criteria like socioeconomic status are controlled for, these populations may experience a lower quality of healthcare than their counterparts [4, 5]. For example, during the COVID-19 pandemic, there was a disproportionately high rate of infection and mortality among racialized and immigrant populations with lower educational levels compared to the general population [6, 7]. Furthermore, stigmatization and historical mistreatment of racialized populations often impact their willingness to engage in health seeking behaviors, which can further exacerbate health inequities [1]. The experiences of underrepresented populations are often rooted in broader sociocultural factors that must be recognized if health disparities are to be addressed [8, 9].

In recent years, implementation researchers acknowledged the importance of incorporating intersectionality, anti-racism, and equity lenses in the design and implementation of interventions and programs [1, 8, 9]. Implementation science, also known as knowledge translation, is *a dynamic and iterative process that includes the study of synthesis, dissemination, exchange, and the ethically-sound application of evidence-based knowledge to improve the health of the population by providing more effective health services and products* [10]. While individuals’ demographic characteristics are important to consider when designing and implementing health interventions, there has been limited discussion regarding the broader social implications of intersecting demographic characteristics as they relate to implementation science [1]. Successfully addressing health inequities is a complex endeavor; it is not sufficient to describe equity gaps in terms of demographic characteristics, such as sex or race alone. Rather, the process warrants use of an intersectional lens that considers how factors such as race, class, gender, and other individual socio-demographic characteristics overlap and intersect with system structures of power and oppression such as sexism, racism, colonialism, and ableism to shape individual experiences and behavior [11, 12].

Intersectionality explores how intersecting power relations at the individual and system level can impact individual experiences [13]. The term “intersectionality” was coined by the Black legal scholar, Kimberlé Crenshaw in 1989 [14]; in her essay, Crenshaw argued that segmenting the dimensions of discrimination paradoxically reinforced the subordination of African American women [14]. Crenshaw proposed the concept of intersectionality as a legal tool to be used in courts and in 1990, sociologist Patricia Hill Collins introduced the concept of intersectionality to sociology and other social science disciplines [15]. Intersectionality offers researchers and clinicians theoretical explanations related to variability in how individuals might experience a situation based on intersecting sociodemographic characteristics in relation to power structures [12]. However, the concept of intersectionality also moves beyond just demographic characteristics (e.g., race, class, gender, sexuality, nationality, ethnicity, ability, age) and intersecting power relations. The focus of intersectionality also includes social location of individuals and groups within intersecting power relations to shape their experiences within and perspectives on the social world and in solving social problems within a given local, regional, national, or global context [16]. Consider the example of a white, older woman who has experienced certain privileges due to her cultural identity (e.g., skin color), but is now feeling excluded or oppressed by society due to the social structures that impact older adults (i.e., ageism). At one point, this individual may have felt very privileged, but may now be struggling to understand why they feel excluded. Our intersecting categories may change with time affecting our feelings of privilege and oppression. Another example of how cultural and social identities play a role in the concept of intersectionality is the example of an Indigenous woman who fears calling the local police for intimate partner violence. Systemic racism contributes to barriers that may prevent her from seeking help after an incident due to cultural barriers to access resources, inaccessible supports and services, and mistrust in the police, criminal justice system, and institutions [17–20]. The application of intersectionality is complex and wide ranging [21]. Intersectionality can be applied within or across disciplines. It can also be applied in a way that captures power dynamics beyond individual identities such as structural social justice or understanding colonialism through an intersectional lens [21, 22].

Tenets of intersectionality suggest that: (1) social identities (e.g., race, gender) are multidimensional, complex, interdependent, and mutually constitutive (2) structures of power, privilege and oppression also interrelate and when interacting with one’s intersecting identities, can impact individual experience (like health) and, (3) the focus of both the theory and practice of intersectionality must be on social justice [23, 24]. Intersectionality requires a new way of analyzing demographic data, which focuses less on the differences between mono-categorical thinking of race or gender, and more on the relationship between categories within power structures, social location, and social problems [25].

The focus on power and social justice in intersectionality includes health inequities that can emerge through inequities in structural power and privilege [26]. For example, racialization and socioeconomic status can separately lead to perceived discrimination in healthcare; however they can also have an intersecting effect [27]. A focus on intersectionality can help to identify health inequities and support the development of more equitable policies and practices in healthcare [26, 28].

Theories, models, and frameworks often guide implementation research that facilitate the uptake and implementation of research into practice [29, 30]. While theories, models, and frameworks can be useful in implementation research, they often do not consider important factors and experiences that impact healthcare inequity [31]. Currently, there are few theories, models, or frameworks to guide implementation researchers and practitioners to use intersectional considerations in their work [32, 33]. While intersectionality methodologies may be applied to each phase of the implementation process, the challenge for the field is to identify how existing theories, models, and frameworks can be operationalized to integrate intersectionality in a way that advances the science and practice for those designing and delivering interventions. As part of a larger project, our goal was to supplement commonly used theories, models, and frameworks in implementation science with an intersectional lens with the goal of designing more equitable programs and addressing health disparities [34]. In this manuscript, we describe our methods to supplement the Consolidated Framework for Implementation Research (CFIR) with an intersectional lens and provide a tool support its use.

## Methods

This work was part of an overarching initiative led by a framework committee (n = 17), which comprised five implementation science developers, two implementation science trainees, five theory, model, and framework experts, four individuals with training in intersectionality, and a critical feminist scholar [35]. We define implementation researchers as individuals who seek to understand real-world circumstances rather than trying to control the environment to remove influence as a causal effect. Implementation practitioners are professionals who support implementation practices and build implementation capacities within a service organization or system.

Ten framework committee meetings, three subgroup meetings and three subgroup review rounds took place between June 2018 and February 2019 to develop the intersectionality supplemented CFIR and its corresponding tool.

Incorporating an intersectional lens into an already established framework requires ongoing iterations and reflexivity. We previously described our methodological approach to prioritizing theories, models, and frameworks for supplementation elsewhere [34, 35]. Briefly, our team began by prioritizing stages in the Knowledge to Action model for optimization using intersectional considerations. Our team established a consensus on a subset of stages within the Knowledge to Action model to focus upon for intersectionality supplementation [34, 35]; the Knowledge to Action model provides an approach to build on the commonalities found in planned action theories [29]. Stages of the Knowledge to Action model are commonly operationalized using theories, models, or frameworks [29]. Via a consensus process guided by the Theory Comparison and Selection Tool (T-CaST) [36] and a nominal group technique, the framework committee prioritized the following stages of the Knowledge to Action model as key stages that would benefit from an intersectional lens: Stage one: identify the knowledge-to-action gap, Stage three: assess barriers to and facilitators of knowledge use, and Stage four: select, tailor, and implement interventions. Next, the framework committee systematically selected common theories, models, and frameworks to operationalize each of the three selected stages from the Knowledge to Action model [34, 35]. Using a nominal group technique, the framework committee reviewed 160 theories, models, and frameworks identified in a comprehensive scoping review on Knowledge Translation and Implementation Science theories, models and frameworks; and prioritized the models and frameworks that were commonly used in implementation science [37]. This review process began with a survey (guided by committee member’s input and the T-CaST tool) to determine the criteria for prioritizing the 160 theories, models, and frameworks. In person and teleconference discussions focusing on how theories, models and frameworks meet intervention developers’ and users’ needs; and survey results were facilitated. The items with the highest median ratings and coverage across key T-CaST criteria (usability, acceptability, and applicability) were selected for consideration for the criteria to use for prioritizing the theories, models, and frameworks. Four smaller groups were then created from the larger committee group; each was assigned 33 theories, models, and frameworks to analyze in relation to the T-CaST prioritization criteria. Members from each group prioritized theories, models, and frameworks for each Knowledge to Action stage using a modified Delphi approach involving two rounds. A final, majority vote was conducted via video conference to select the models and frameworks best suited to operationalize the Knowledge to Action model. The three models and frameworks selected to operationalize three stages of the Knowledge to Action were: (1) the Iowa model of evidence-based practice for Stage 1: identify the knowledge-to-action gap, (2) the Consolidated Framework for Implementation Research (CFIR) for Stage 3: assess barriers to and facilitators of knowledge use, and (3) the Theoretical Domains Framework (TDF) for Stage 4: select, tailor and implement interventions. The supplementation of the Iowa model [35] and the TDF [34] using intersectional considerations are described elsewhere. This paper will focus on supplementing CFIR using an intersectional lens.

CFIR is a common conceptual framework that can guide the collection, coding and analysis of data to comprehensively understand contexts that may influence intervention implementation and effectiveness [38]. CFIR is commonly used to plan, implement, and evaluate interventions in various contexts and settings [38]. It draws on 19 theories, models, and frameworks to provide a meta-framework for implementation research and is composed of five domains and 26 constructs [38]. The domains include: intervention characteristics (eight constructs), outer setting (four constructs), inner setting (five constructs), individual characteristics of individuals (five constructs) and process of implementation (four constructs) (see Appendix A) [38].

### CFIR subgroup structure and processes

The principal investigator (SES) contacted the developer of CFIR to describe our team’s intent to supplement the framework and to ensure our group was using the most recent version of CFIR. After it was confirmed that we were using the most recent version of CFIR, we assembled a subgroup from the framework committee (n = 17), called the CFIR subgroup (n = 7), which henceforth will be referred to as the subgroup. We chose to engage a subset of the framework committee to facilitate conversations and support interactivity between all members. The subgroup was composed of one implementation science researcher, two implementation science practitioners, two implementation science researcher-practitioners, one implementation science trainee, and an intersectionality expert. Subgroup members were from Alberta, British Columbia, and Ontario. Of the seven members in the subgroup, most identified as white, heterosexual, and female. Most also reported that they were married, had a masters or doctoral degree, lived in a large population center in their own home, were employed full time, and had an annual family income >$120,000 CAD.

### Step one: prioritizing CFIR domains for supplementation

The subgroup held one review round over email to prioritize the domains and constructs for supplementation and then two virtual meetings to discuss the results of the prioritization process. We asked the subgroup to individually brainstorm how they would rank each domain in CFIR using the following criteria: ‘very high priority’, ‘high priority’, ‘neutral’, ‘low priority’, or ‘very low priority’ in terms of intersectional considerations. In addition, the subgroup was asked to consider: (1) “how would you think about conducting a facilitator of and barriers to assessment for each CFIR construct”; and (2) “how would you think about intersectionality and intersecting categories for each CFIR construct”. After one week of brainstorming, the subgroup met via video conference to discuss and identify the CFIR domains and constructs related to stage three (assessing barriers and facilitators to knowledge use) of the Knowledge to Action model. The discussion was facilitated by an experienced research coordinator (DK). The discussion began with the facilitator asking the group if they considered the outer setting domain important to incorporate intersectional considerations; each domain and construct was discussed and ranked using the prespecified criteria. After the discussion, the facilitator circulated a table to the subgroup via email and asked each of the seven members to rank each domain and construct as ‘very high priority’, ‘high priority’, ‘neutral’, ‘low priority’, or ‘very low priority’. The subgroup then met via teleconference to discuss these responses and finalize the domains and constructs that were to be supplemented. We discussed domains and constructs marked as ‘very high’ or ‘high’ priority by the critical feminist scholar, even if the other members of the subgroup ranked it ‘neutral’ or lower. Through this discussion, group consensus was established for the priority level of each domain. Domains ranked ‘very high priority’ and ‘high priority’ were supplemented with intersectional considerations. The subgroup strived for consensus on which domains to enhance with an intersectional lens. All subgroup members had a chance to voice their thoughts in meetings or by email.

### Steps two and three: developing the intersectionality supplemented CFIR and the CFIR tool

Following the prioritization of the CFIR constructs, the subgroup met once each via video conference and teleconference to develop the intersectionality prompts/reflection questions for each domain and construct using the ‘cfirguide.org’ definitions as a starting point. The subgroup used a collaborative and iterative approach to draft and refine a tool to include intersectionality-supplemented definitions for each prioritized construct, with prompts and reflections to aid other researchers when using the supplemented framework. Following the development of the initial tool, the subgroup conducted two rounds of revisions over email to iteratively edit the tool. The framework committee (n = 17) then reviewed the tool and the research project support team integrate this feedback. Lastly, the project support team developed graphics to optimise the usability of the supplemented framework.

## Results

We reached consensus on applying an intersectional lens to 26 constructs within the five original domains of the CFIR. Through the consensus process we added two additional constructs (i.e., total of 28 constructs within five domains) to further enhance the CFIR for use with an intersectional lens; we added “outer systems and structures” and “outer cultures” to the outer setting domain. We also added intersectionality prompts to 13 out of the 28 constructs.

### Step one: prioritizing CFIR domains for supplementation

In the first subgroup meeting, participants identified three CFIR domains that they believed were relevant to stage three of the Knowledge to Action model including (1) outer setting; (2) inner setting, and (3) characteristics of individuals. The “characteristics of individuals” domain was identified as the most important and the subgroup highlighted that the ‘other personal attributes’ construct within the “characteristics of individuals” domain was critical to supplement with an intersectional lens. The “implementation process” domain was deemed not relevant to stage three because the subgroup believed it was more relevant to stage four (“selecting, tailoring, and implementing interventions”) of the Knowledge to Action model.

These results were discussed during the second meeting. There was low level of agreement for inclusion of several CFIR constructs for the following domains: outer setting (external policies and incentives), inner setting (access to knowledge and information), and characteristics of individuals (individual identification with organization) (Table 1).

**Table 1:** Priority assessment for CFIR intersectionality considerations

Consolidated Framework for Implementation Research domain and constructs*	Priority level
**1. Outer setting**	
1.1. Patient needs and resources	High
1.2. Cosmopolitanism	Low
1.3. Peer pressure	Medium
1.4. External policies and incentives	High**
**2. Inner setting**	
3.1. Structural characteristics	Low
3.2. Networks and communications	High
3.3. Culture	High
3.4. Implementation climate	Low to medium
3.4.1. Tension for Change	Low to medium
3.4.2. Compatibility	High
3.4.3. Relative Priority	Medium
3.4.4. Organizational Incentives and Rewards	High
3.4.5. Goals and Feedback	Low to medium
3.4.6. Learning Climate	High
3.5. Readiness for implementation	Low to medium
3.5.1. Leadership Engagement	Low to medium
3.5.2. Available Resources	Medium
3.5.3. Access to Knowledge and Information	Medium to high**
**4. Characteristics of individual**	
5.1. Knowledge and beliefs about the intervention	High
5.2. Self-efficacy	High
5.3. Individual stage of change	Medium
5.4. Individual identification with organization	Low**
5.5. Other personal attributes	High

*The intervention characteristics and implementation process domains were excluded due to lack of relevance

** Determined during third meeting

The constructs with low levels of agreement were further discussed at the third meeting. These constructs included ‘external policies and incentives’ [outer setting]; ‘access to knowledge and information’ [inner setting]; and ‘individual identification with organization’ [characteristics of individuals]. The subgroup agreed that “external policies and incentives” (from the outer setting domain) and “access to knowledge and information” (from the inner setting domain) should be considered high priority for intersectional considerations, while “individual identification to organization” (from the characteristics of the individual domain) should be considered as a low priority. The subgroup believed the “individual identification to organization” was already captured in other constructs such as “compatibility”. The subgroup also discussed the need to include the construct of “culture” in the outer setting domain; in this context, culture was defined as the norms, values, and basic assumptions of a given society. They felt it was important to distinguish between inner culture (within the inner setting domain) and outer culture (within the outer setting domain). The inner setting domain includes “characteristics of the implementing organization such as team culture, compatibility and relative priority of the intervention, structures for goal-setting and feedback, leadership engagement, and the implementation climate” while outer settings are the “external influences on intervention implementation including patient needs and resources, cosmopolitanism or the level at which the implementing organization is networked with other organizations, peer pressure, and external policies and incentives” [39]. The subgroup also discussed potential limitations of the “other personal attributes” construct in the “characteristics of individual” domain since the “other personal attributes” construct was perceived to overlook a wide range of intersectional concepts. The subgroup’s preference was to emphasize the need to address structural barriers that impact individual experience rather than underscore personal attributes as contributors to individual’s experiences. As a result, the subgroup suggested adding an “outer structures and systems” construct to the outer setting domain to distinguish between personal and structural characteristics. The subgroup flagged the importance of explicitly highlighting structural and attitudinal barriers such as sexism, racism, anti-Indigenous structures, and colonialism in the “outer structures and system” construct.

### Steps two and three: developing the intersectionality supplemented CFIR and tool

**Fig. 1:**
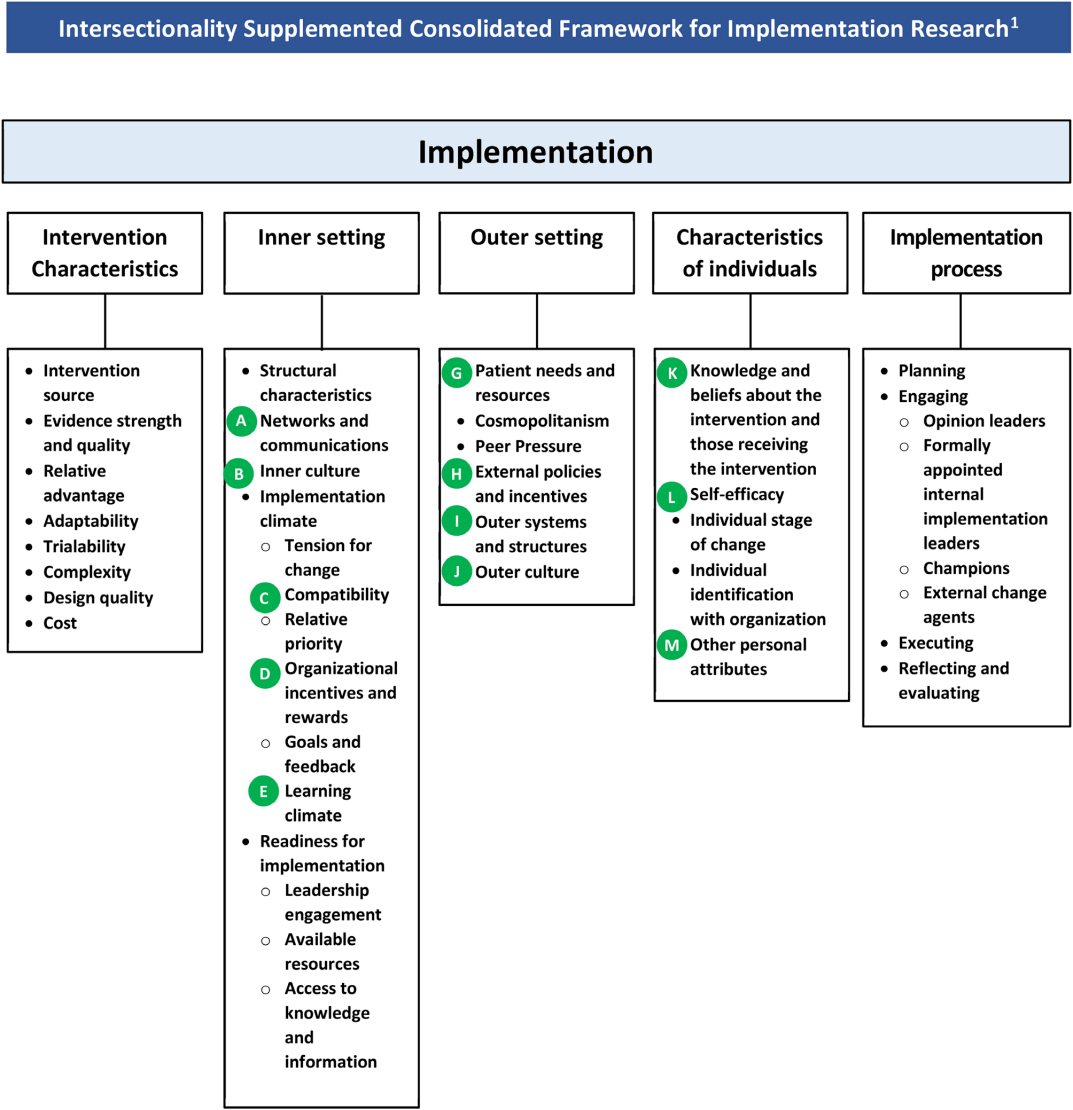
The intersectionality supplemented Consolidated Framework for Implementation Research. The green dots denote prompts and reflection points for researchers when determining how to incorporate intersectionality considerations into a research study

Steps two and three consisted of two review rounds via email. Members of the subgroup individually reflected on the high priority constructs identified in the previous three meetings and then collectively modified the intersectionality supplemented CFIR through multiple email communications. The major changes included providing: (1) explanations on why certain constructs were deemed high priority; and (2) a clear definition on changes to the two “culture” constructs (i.e., “inner culture” in the inner setting domain and the “outer culture” in the outer setting domain). In addition, the subgroup developed visualizations for the intersectionality supplemented definitions and the intersectionality prompts for the intersectionality supplemented CFIR tool. In Fig. 1, we present the intersectionality supplemented CFIR, which includes the five original domains and 28 constructs; we added two constructs to the outer setting domain (i.e., “outer systems and structures” and “outer culture”). Table 2 compares the original CFIR to the intersectionality supplemented CFIR.

**Table 2:** CFIR domains and construct descriptions (original and intersectionality supplemented CFIR comparison)”

CFIR Domain/ Construct	Domain/Construct Description (for more information, visit: https://cfirguide.org/constructs/)	Intersectionality supplemented Domain/Construct Description
**CFIR Domain: Outer setting**	The ‘economic, political, and social context within which an organization resides.’	The economic, political, geographical, environmental, physical, environmental, and social context within which an organization resides.
Patient needs and resources	The extent to which patient needs, as well as barriers and facilitators to meet those needs, are accurately known and prioritized by the organization.	The extent to which diverse patient perspectives, values, needs, as well as barriers (e.g., historical distrust of medical systems) and facilitators (e.g., high socioeconomic status) to meet those needs are accurately known, aligned with, and prioritized by the organization
External policies and incentives	A broad construct that includes external strategies to spread interventions, including policy and regulations (governmental or other central entity), external mandates, recommendations and guidelines, pay-for-performance, collaboratives, and public or benchmark reporting.	A broad construct that includes external strategies to spread interventions, including policy and regulations (governmental or other central entity), external mandates, recommendations and guidelines, pay-for-performance, collaboratives, and public or benchmark reporting and that the creation and sustainment of these strategies addresses systems of power, inclusivity, and equity.
Outer structures and systems	N/A (not in CFIR)	The overlapping structures and systems of a given society, including systems of privilege and oppression (e.g., sexism, racism, ableism).
Outer culture	N/A (not in CFIR)	The norms, values, and basic assumptions (e.g., heteronormativity) of a given society.
**CFIR Domain: Inner setting**	The ‘structural, political, and cultural context through which the intervention proceeds’ and the relationship between these elements.	The ‘structural, political, power, and cultural context through which the intervention proceeds’ and the relationship between these elements.
Networks and communications	The nature and quality of webs of social networks and the nature and quality of formal and informal communications within an organization	The nature, quality, and inclusivity of webs of social networks and the nature, quality, and access to formal and informal communications within an organization.
Inner culture	Norms, values, and basic assumptions of a given organization.	Norms, values, power structures, and basic assumptions (e.g., heteronormativity) of a given organization.
Compatibility (sub-construct to CFIR construct “Implementation climate”)	The degree of tangible fit between meaning and values attached to the intervention by involved individuals, how those align with individuals’ own norms, values, and perceived risks and needs, and how the intervention fits with existing workflows and systems.	The degree of tangible fit between individuals’ intersecting social categories and the meaning and values attached to the intervention by involved individuals, how those align with individuals’ own norms, values, ways of knowing, and perceived risks and needs, and how the intervention fits with existing workflows and systems.
Organizational incentives and rewards (sub-construct to CFIR construct “Implementation climate”)	Extrinsic incentives such as goal-sharing awards, performance reviews, promotions, and raises in salary, and less tangible incentives such as increased stature or respect.	Existence of and access to extrinsic incentives such as goal-sharing awards, performance reviews, promotions, and raises in salary, and less tangible incentives such as increased stature or respect.
Learning climate (sub-construct to CFIR construct “Implementation climate”)	A climate in which: (a) leaders express their own fallibility and need for team members’ assistance and input; (b) team members feel that they are essential, valued, and knowledgeable partners in the change process; (c) individuals feel psychologically safe to try new methods; and (d) there is sufficient time and space for reflective thinking and evaluation.	A climate in which: (a) leaders, representative of diverse intersecting social factors, express their own fallibility and need and respect for team members’ assistance and input; (b) team members, representative of diverse intersecting social factors, feel they are partners and that their perspective is encouraged, essential, heard, valued, and considered knowledgeable in the change process; (c) individuals feel psychologically safe to try new methods; and (d) there is sufficient time and space for reflective thinking and evaluation in multiple venues/means (e.g., written reflection, discussion).
Access to knowledge and information (sub-construct to CFIR construct “Readiness for implementation”)	Ease of access to digestible information and knowledge about the intervention and how to incorporate it into work tasks.	Ease of access to digestible information and knowledge about the intervention and how to incorporate it into work tasks.
**CFIR Domain: Characteristics of Individuals**	The individuals responsible for carrying out the intervention or otherwise related to the intervention, their agency, and their relationships to each other and the intervention.	The individuals responsible for carrying out the intervention or otherwise related to the intervention, their agency, intersecting social categories, and their relationships (e.g., power dynamics) to each other, the intervention, and those impacted by the intervention (e.g., patients).
Knowledge and beliefs about the intervention and those receiving the intervention	Individuals’ attitudes toward and value placed on the intervention as well as familiarity with facts, truths, and principles related to the intervention.	Individuals’ attitudes toward and value placed on the intervention as well as familiarity with and access to facts, truths, and principles related to the intervention and those receiving the intervention (e.g., patients, other health providers).
Self-efficacy	Individual belief in their own capabilities to execute courses of action to achieve implementation goals.	An individual’s belief in their own capabilities (related to their intersecting social factors) to execute courses of action to achieve implementation goals.
Other personal attributes	A broad construct to include other personal traits such as tolerance of ambiguity, intellectual ability, motivation, values, competence, capacity, and learning style.	A broad construct to include the intersection of other personal traits and social factors such as tolerance of ambiguity, motivation, values, competence, learning style. These individual traits and social factors interact with each other and other domains including the outer and inner setting (e.g., one’s values regarding educational achievement will be influenced by social systems, such as sexism and racism).

One subgroup member suggested incorporating a set of reflection questions or prompts for intervention developers to improve usability of the intersectionality supplemented CFIR. As a result, the subgroup developed instructions for use and background information on tool development. Project support staff then developed graphics to support usability of the tool; notably, we aimed to reflect graphics and language consistent with the original CFIR publication as the original definitions were perceived to be most familiar to end users. In addition, the subgroup confirmed the addition of the “outer systems and structures” construct in the outer setting domain of the supplemented framework. Intersectionality prompts were added to 13 of the 28 constructs. Table 3 summarises the prompts that were developed.

**Table 3:** Reflective prompts using the intersectionality supplemented Consolidated Framework for Implementation Research

CFIR Domain/ Construct	Intersectionality Prompts for intersectionality supplemented Domain/Construct
**CFIR Domain: Outer setting**	• How did the current structure (economic, political, geographical, environmental, structural (e.g., economic) and social context of the outer setting come to be (e.g., economic downturns, ideologies of government leaders, legal precedent)? • How have different spheres (e.g., economic, political, and environmental) intersected to produce the current outer setting?
Patient needs and resources	• Do we accurately and comprehensively understand the diverse patient experience related to this intervention? • How might a patient’s intersecting categories influence their experience related to this intervention? • How have diverse patient perspective, values, needs, and voices been incorporated by the organization? • How might previous work to integrate patient’s perspectives, values, and needs influence this intervention?
External policies and incentives	• To what extent are external strategies (e.g., policies, regulations, mandates, recommendations and guidelines, reporting) non-discriminatory and address institutional forms of marginalization (e.g., racism, sexism, ageism)? • How might institutionalized forms of marginalization (e.g., racism, sexism, and ageism) influence the success of the intervention? • To what extent are external strategies (e.g., policies, regulations, mandates, recommendations and guidelines, reporting) inclusive? • What assumptions do external strategies make about those expected to change their behavior in the intervention (e.g., forms of (dis)ability, gender roles)? • Do external strategies and incentives reinforce stereotypes? If stereotypes are reinforced, how might this influence the success of the intervention? • Do all individuals and organizations have equal access to external strategies and incentives? How might this access influence the success of the intervention?
Outer structures and systems	• What systemic forms of oppression exist (e.g., sexism, ableism) within institutions? Who holds power in institutions? • How are populations related to the intervention portrayed in the media? • What structural inequities exist within the health area or population the intervention impacts?
Outer culture	• What assumptions does the society or community (outside the organization) make about those expected to change their behavior in the intervention (e.g., what assumptions does society make about the emergency room nurses expected to deliver a new questionnaire on fall prevention)? • How might societal biases (hidden and overt) influence knowledge use? For example, does society respect the role of all health professionals equally (e.g., physiotherapists, nurses, physicians, physician assistants)? • How might the roles that individuals are expected to play within society (e.g., gender roles) influence knowledge use? • How does society view patients that are expected to use or be affected by knowledge use (e.g., what biases does society hold regarding older adults)?
**CFIR Domain: Inner setting**	• Who holds power within the organization? What intersecting categories do they represent? Are they similar intersecting categories to those whose behavior is targeted for change? • How may these power relations affect the implementation intervention (positively and/or negatively)?
Networks and communications an organization.	• How might the principle of homophily (i.e., birds of a feather flock together) influence who has access to information and who does not? How might access to information impact the success of the intervention? • Who are the leaders (formal and informal) at the organization? What intersecting categories do they represent? Are these leaders representative of the intersecting categories of those individuals expected to change their behavior? • How might power structures influence informal communications? How might these communications affect the intervention? • How does the organization support different ways of communicating? How can different ways of communicating support the success of the intervention? • This includes physical access to communications (e.g., computers) and accessibility of communications (e.g., website meets accessibility standards). • How might social structures influence informal communication systems? How might these informal communication systems impact the success of the intervention?
Inner culture	• What assumptions does the organization make about its staff? How might these assumptions influence the intervention? • What assumptions does the organization make about the population it supports? How might these assumptions about the population influence the intervention? • What are the values of the organization? How might these values influence the success of the intervention? • How are projects prioritized? How might the prioritization process influence the intervention? • What biases does the organization hold (hidden and overt)? Do these biases align, reinforce, or counter biases in the outer culture(s) (i.e., in broader society)? For example, does the organization assume the older adults are not active and frail?
Compatibility (sub-construct to CFIR construct “Implementation climate”)	• Does the intervention align with the values, norms, ways of knowing, and existing workflow of those changing their behavior? • What assumptions are being made about the abilities of those expected to change their behavior? • What assumptions are being made about the workflow of those expected to change their behavior?
Organizational incentives and rewards (sub-construct to CFIR construct “Implementation climate”)	• Does everyone in the organization have the same access to organizational incentives and rewards? How might this reward access impact the success of the intervention? • Are there hidden or overt biases towards those who attain formal (e.g., promotions) or informal (e.g., stature or respect) organizational rewards? How might these biases influence the success of the intervention? • Is the range of values and preferences of those whose behavior we are trying to change considered when establishing incentives? (i.e., do all people value the proposed or available incentives and rewards)?
Learning climate (sub-construct to CFIR construct “Implementation climate”)	• How do leaders in the organization display vulnerability or considerations of power re-distribution? How might this leadership behavior influence the success of the intervention? • Do all individuals expected to change their behavior have equitable access to sufficient time and space for reflective thinking and evaluation in multiple venues/means (e.g., do part-time staff or those who work from home have protected time for reflection)?
Access to knowledge and information (sub-construct to CFIR construct “Readiness for implementation”)	• Does everyone involved in the intervention have access to information in a format that works for them? • Have individual representatives of different user groups contributed to the creation and dissemination of the knowledge? • Has the source of information been critically appraised by a diverse group of people occupying diverse intersecting categories? • How does literacy, health literacy, ehealth literacy, vision, numeracy, impact access and digestibility of information about the intervention? • Does the knowledge use universally understood analogies? • Avoid using culture-specific analogies (e.g., sports terminology such as “hit a home run”)? • Avoid using potentially offensive or triggering language (e.g., “in the trenches”).
**CFIR Domain: Characteristics of Individuals**	• What assumptions do individuals expected to change their behavior make about those affected by the intervention (e.g., what do providers assume about patients in precarious housing situations)? • What assumptions are made about the agency of those expected to change their behavior? What do they have control over? • How might the categories of the individuals expected to change their behavior intersect? How might these intersecting categories affect individuals’ interactions with others?
Knowledge and beliefs about the intervention and those receiving the intervention	• How might an individual’s intersecting categories (e.g., age, education, gender) influence their access to the facts, truths, and principles related to the intervention? • How might an individual’s intersecting categories influence their knowledge and beliefs toward an intervention? • How might an individual’s intersecting categories affect the value placed on the intervention in comparison to competing priorities?
Self-efficacy	• What, beyond an individual, may impact their self-efficacy (e.g., gender stereotypes)? • How might an individual’s intersecting categories impact their self-efficacy to execute the intervention? • How might systems of oppression (e.g., racism, sexism, and ageism) affect individuals’ self-efficacy to deliver the intervention?
Other personal attributes	• Reflect on our assumptions of what attributes we classify as modifiable by an individual. What external influences, beyond the individual, may be influencing these attributes? • Think broadly: what intersecting categories and personal dimensions may influence the intervention? What is the relationship between these categories? • Are the categories conceptualized in an additive or multiplicative way (e.g., values + learning style) or are they conceptualized as connected? Focus on the interdependencies and mutual constitution of these categories as opposed to considering them as independent categories. • For those expected to change their behavior, what intersecting categories may be most influential (e.g., the intersection of values and tolerance for ambiguity)? • How may an individual’s life experiences shape the traits (e.g., education) that enable them to engage in the target behavior?

## Discussion

Our interdisciplinary team aimed to supplement the CFIR to incorporate intersectional considerations. Following three subgroup meetings and several rounds of iterative revisions, the resulting intersectionality supplemented CFIR includes the five original domains with two additional constructs for a total of 28 constructs. Intersectionality prompts were added to 13 of the 28 constructs. We included several considerations and prompts to help researchers reflect on how individual identities and structures of power may play a role in implementing evidence-based interventions.

Intersectionality is an analytic tool – a way of thinking about identity and its relationship to power [26]. Originally articulated by Black feminists to describe the experiences of Black women, intersectionality has brought to light the importance of considering the compounding of individual characteristics with the systems of oppression and privilege [40]. Numerous academics have explored the value of bringing an intersectional perspective to empirical research, and as a result, recommendations for integrating intersectionality in qualitative research have been proposed [40–43]. However, the employment of quantitative methodologies with an intersectional approach have been heavily criticized by intersectionality scholars, who emphasize the dangers of additive, single-axis thinking [40–43]. For example, Bowleg and colleagues argue that the notion of social identity and social inequality based on ethnicity, sexual orientation, sex, gender, among other characteristics, are intersectional rather than additive [42–44]. The authors argue that a key dilemma for intersectionality researchers is that the additive assumption (e.g., Black + Lesbian + Woman) is inherently distinct from the intersectional lens (e.g., Black Lesbian Woman) [44]. The term intersectionality continues to be named, but not deeply embedded in research, particularly in implementation research, which is a critical gap given the massive health inequities that exist worldwide.

Our intersectionality supplemented CFIR offers recommendations for considering intersectionality at various stages of the implementation process. The reflection prompts in Table 3 consider each construct in the original CFIR and attempt to operationalize these with intersectional considerations. We specifically selected definitions and prompts that could be applied in research. The prompts are meant to guide researchers, thinking processes rather than be copied and pasted into an interview guide. Our considerations and prompts for the CFIR are designed to assist researchers in asking questions about intersecting, interdependent, and mutually constitutive experiences without resorting to an additive approach. Researchers are responsible for interpreting data in the societal context that it was collected from regardless of whether qualitative or quantitative approaches are used [44]. Thus, asking questions within the context of the sociohistorical and structural society can provide insight into such constructs. We recognise that as research advances in this area, additional revisions may be required to reflect the evolving understanding of using an intersectionality lens in research.

One example of where intersectional considerations may be helpful is when considering the “patient needs and resources” construct [outer setting domain]; for example, a study identified that cervical screening rates among South Asian Muslim immigrants in Canada were much lower compared to women born in Canada [45]. The research reported that lack of knowledge about cervical cancer, transportation, and language were barriers to screening; however, considering intersecting categories of religion and education may have prompted different interview questions and a better understanding of what patients deemed important and what system changes needed to happen. The coexistence of implementation and intersectional considerations also launches the potential to examine interesting questions regarding interactions between the dimensions of oppression and privilege across different levels [41]. The use of intersectional considerations in implementation science are still in its infancy, but we predict such considerations will have a meaningful and profound impact on our healthcare system as they shift the focus from individual level change to system change, which is needed to tackle health inequities.

After identifying a research question, the qualitative research process involves choosing a framework or theoretical lens (e.g., phenomenology, grounded theory, ethnography), a methodology (e.g., observation, case study), and a data collection technique (e.g., focus groups, photographs). When utilizing the intersectionality supplemented CFIR, researchers should consider when and how they intend to incorporate intersectionality into their study. For example, researchers can decide whether to use our supplemented framework to guide the entire study process or incorporate the updated framework into the data analysis stage (e.g., mapping of facilitators and barriers using CFIR). The supplemented CFIR can also be used to guide the interview process by explicitly asking about barriers such as how historical distrust of the medical system may affect uptake of an intervention (see Table 2 for specific intersectionality constructs). We recommend that intersectionality be considered during the study conceptualization phases as the constructs supplemented in our framework can generate important considerations for interactions with participants during the recruitment, data collection, analysis, and dissemination phases.

In implementation science, researchers often need to assess context. CFIR is commonly used to assess such context; however the CFIR is a framework, not an assessment tool [38]. Usually, researchers use the CFIR to operationalize a method of assessment; for instance, using the CFIR technical assistance website to transform domains into surveys, develop an interview guide, or categorize interview data. The way researchers phrase questions shape how participants respond to them and a pivotal aspect of asking good questions is to understand intersecting categories in relation to power structures [44]. It is also important to reflect on who is asking and guiding the interview questions (e.g., is there a power dynamic between the interviewee and interviewer?). Typically, not all domains and constructs are utilized when using the original CFIR [37]. Similarly, we recognize that it may not be feasible to consider all 15 prompts alongside standard operationalizations of the CFIR. Instead, we recommend that users prioritize prompts that they consider will be useful and relevant to their study. Additionally, researchers should reflect on how power and privilege operate within themselves, their research study team, and research organization as this can affect stakeholder relationships and collaboration [46]. For example, the growing lexicon of academic language that privileges researchers can become an oppressive and exclusionary factor for populations of focus, especially in implementation science where the field is growing at an exponential rate [46]. Our intent was not to replace the original CFIR, but rather to provide researchers with an additional lens.

An important consideration is the introduction of outer systems and culture as constructs in our supplemented CFIR tool. Outer culture is a broader based determinant of health that acts at the community, population, and national level [47]. There is growing recognition of the need for culturally safe, patient-centered care to improve health outcomes, particularly among minority populations [47]. Health practitioners, healthcare organizations, and healthcare systems need to engage towards culturally safe environments; to do this they (i.e., individuals in power) must be prepared to critique the power structures and challenge their own culture and culture systems [48]. The prompts proposed in our supplemented CFIR tool may help researchers challenge their own ways of thinking to possibly improve the quality of the information gained when conducting surveys or interviews guided by CFIR. Continued neglect of social considerations, as well as the larger systemic power structures in which the social considerations are embedded, may result in missed opportunities for effective implementation. As a result, lack of intersectional considerations may perpetuate future systematic health inequities. The explicit use of CFIR with the greater application of intersectional considerations within implementation science has the potential to improve researchers’ collective abilities to more specifically document inequalities within intersectional groups.

### Strengths and limitations

Our study has several strengths. First, we strove to build a team of practitioners and implementation science users from across Canada with various expertise in implementation science and intersectionality. We also engaged with implementation science users who were not yet familiar with intersectional concepts, which we believe helped create a tool that was potentially more accessible to the novice researcher or practitioner. We also considered accessibility limitations, and so, we engaged in multiple video conferences and teleconferences. Lastly, our comprehensive and rigorous approach is consistent with other tool development methods reported in implementation science [36].

Our methods also had limitations. As described, we engaged with implementation science users who, were not yet familiar with intersectionality concepts. To reduce this limitation, we created small group discussions of no more than five individuals to review concepts. We also held capacity building sessions on intersectionality led by experts in the areas of implementation science and intersectionality. Nevertheless, it is possible that a group of different interdisciplinary researchers may have prioritized a different set of theories, models, and frameworks for intersectionality enhancements. We also recognize that those involved in the project represent a limited range of privileged identities and may affect the generalizability of the results. Furthermore, we recognize that biases may have influenced our approach, due to the lack of representation of historically marginalized social identities in our subgroup. In efforts to limit these biases, we drew upon works and guidance authored by individuals from marginalized groups to inform our decision making [49]. Future research can further build on the intersectionality categories presented to develop tailored, culturally-relevant prompts and interventions for subsets of marginalized groups (e.g., Indigenous considerations). Lastly, this work was completed prior to the publication of the updated CFIR [46]; however the principles of intersectionality outlined in this paper can be applied to the updated framework.

### Future directions

This project is part of a larger program of research. The next steps are to test the usability of these tools with implementation scientists, researchers, and clinicians, and then pilot the tools under real-world conditions. We do not expect that the supplemented framework and tool alone will change behavior. During the pilot trial, we will aim to understand the facilitators of and barriers to using the intersectionality supplemented CFIR and tool in practice. In addition, it is recommended that future research build on the intersectionality categories presented to develop tailored, culturally relevant prompts and interventions for subsets of marginalized groups.

## Conclusion

After several iterative discussions with an expert panel, we developed the intersectionality supplemented CFIR, which aims to support implementation researchers and practitioners to consider the context of privilege and disadvantage in their work, rather than the study of individual demographic characteristics alone. We also developed a set of prompts and reflection considerations that can be used by implementation intervention developers and researchers to embed intersectionality into research projects.

### Electronic supplementary material

Below is the link to the electronic supplementary material.

Supplementary Material 1

Supplementary Material 2

## Data Availability

All data generated or analysed during this study are included in this published article and its supplementary information files (Supplementary File 1: Appendix A; Supplementary File 2: CFIR Subgroup Intersecting Categories Survey Results).

## Abbreviations

CFIR: Consolidated Framework for Implementation Research
T-CaST: Theory Comparison and Selection Tool

## References

[1] Baumann AA, Cabassa LJ. Reframing implementation science to address inequities in healthcare delivery. BMC Health Serv Res. Mar 12 2020;20(1):190. 10.1186/s12913-020-4975-3. 10.1186/s12913-020-4975-3 PMC7069050 32164706

[2] Alvidrez J, Stinson N Jr. Sideways progress in intervention research is not sufficient to Eliminate Health disparities. Am J Public Health. Jan 2019;109:S102–4. 10.2105/AJPH.2019.304953. 10.2105/AJPH.2019.304953 PMC6356126 30699028

[3] Blendon RJ, Schoen C, DesRoches CM, Osborn R, Scoles KL, Zapert K. Inequities in health care: a five-country survey. Health Aff (Millwood). May-Jun 2002;21(3):182–91. 10.1377/hlthaff.21.3.182. 10.1377/hlthaff.21.3.182 12025982

[4] Egede LE. Race, ethnicity, culture, and disparities in health care. J Gen Intern Med. Jun 2006;21(6):667–9. 10.1111/j.1525-1497.2006.0512.x. 10.1111/j.1525-1497.2006.0512.x PMC1924616 16808759

[5] Institute of Medicine (US) (2003). Committee on understanding and Eliminating Racial and Ethnic Disparities in Health Care.

[6] Sundaram ME et al. “Individual and social determinants of SARS-CoV-2 testing and positivity in Ontario, Canada: a population-wide study,“ *CMAJ*, vol. 193, no. 20, pp. E723-E734, May 17 2021, 10.1503/cmaj.202608. 10.1503/cmaj.202608 PMC8177943 33906966

[7] Public Health Ontario. “COVID-19 in Ontario – A Focus on Diversity.“ https://www.publichealthontario.ca/-/media/documents/ncov/epi/2020/06/covid-19-epi-diversity.pdf?la=en (accessed.

[8] O’Neill J, et al. Applying an equity lens to interventions: using PROGRESS ensures consideration of socially stratifying factors to illuminate inequities in health. J Clin Epidemiol. Jan 2014;67(1):56–64. 10.1016/j.jclinepi.2013.08.005. 10.1016/j.jclinepi.2013.08.005 24189091

[9] Mena E, Bolte G, Group AGS. Intersectionality-based quantitative health research and sex/gender sensitivity: a scoping review. Int J Equity Health. Dec 21 2019;18(1):199. 10.1186/s12939-019-1098-8. 10.1186/s12939-019-1098-8 PMC6925460 31864366

[10] Canadian Institute for Health Research. “Knowledge Translation at CIHR.“ https://cihr-irsc.gc.ca/e/29418.html (accessed.

[11] Crenshaw K (1993). Intersectionality, identity politics, and Violence against women of color. Standford law Rev.

[12] Atewologun D. Intersectionality Theory and Practice, Oxf Res Encycl Bus Manag, 2018.

[13] Collins PHSB (2020). Intersectionality. 2nd Edition.

[14] Crenshaw K. “Demarginalizing the Intersection of Race and Sex: A Black Feminist Critique of Antidiscrimination Doctrine, Feminist Theory and Antiracist Politics.,“ *Univ Chic Leg Forum*, vol. 8, 1, pp. 1–31, 1989. [Online]. Available: https://chicagounbound.uchicago.edu/cgi/viewcontent.cgi?article=1052&context=uclf.

[15] Hill Colins P (1990). Black Feminist thought.

[16] Hill Collins DSE, Ergun P, Inger E, Bond F, Martinez-Palacios KD (2021). J “Intersectionality in Critical Social Theory".

[17] Canada Parliament (2020). "Debates of the Senate".

[18] Canada, Parliament. “Debates of the Senate,“ vol. 151, no. 27, 2020.

[19] Missing and Murdered Indigenous Women and Girls., “Reclaiming Power and Place: The Final Report of the National Inquiry into Missing and Murdered Indigenous Women and Girls.,“ 2019.

[20] Pauktuutit Inuit Women of Canada and, Comack E. ““Addressing gendered violence against Inuit women: A review of police policies and practices in Inuit Nunangat.” Report in Brief and Recommendations.,“ 2020.

[21] Carbado DW, Crenshaw KW, Mays VM, Tomlinson B. “INTERSECTIONALITY: Mapping the Movements of a Theory,“ *Du Bois Rev*, vol. 10, no. 2, pp. 303–312, Fall 2013, 10.1017/S1742058X13000349. 10.1017/S1742058X13000349 PMC4181947 25285150

[22] Moradi B, Grzanka PR. Using intersectionality responsibly: toward critical epistemology, structural analysis, and Social Justice activism. J Couns Psychol. Oct 2017;64(5):500–13. 10.1037/cou0000203. 10.1037/cou0000203 29048196

[23] Bowleg L. The problem with the phrase women and minorities: intersectionality-an important theoretical framework for public health. Am J Public Health. Jul 2012;102(7):1267–73. 10.2105/AJPH.2012.300750. 10.2105/AJPH.2012.300750 PMC3477987 22594719

[24] Buchanan NT, Wiklund LO. Intersectionality Research in Psychological Science: resisting the tendency to disconnect, dilute, and depoliticize. Res Child Adolesc Psychopathol. Jan 2021;49(1):25–31. 10.1007/s10802-020-00748-y. 10.1007/s10802-020-00748-y 33400076

[25] Hill BS. CP, Intersectionality. " Polity Press,; 2016.

[26] Bowleg L, “Evolving Intersectionality Within Public Health. From analysis to action. Am J Public Health. Jan 2021;111(1):88–90. 10.2105/AJPH.2020.306031. 10.2105/AJPH.2020.306031 PMC7750585 33326269

[27] Stepanikova I, Oates GR. Perceived Discrimination and Privilege in Health Care: The Role of Socioeconomic Status and Race. Am J Prev Med. Jan 2017;52. 10.1016/j.amepre.2016.09.024. no. 1S1, pp. S86-S94. 10.1016/j.amepre.2016.09.024 PMC5172593 27989297

[28] Cole E, Duncan LE. Better policy interventions through intersectionality, Social Issues and Policy Review, 2022.

[29] Graham TJ, Gagnon ID. M, *Knowledge Translation in Healthcare. Second Edi*. 2013, pp. 75–92.

[30] Grimshaw JM, Eccles MP, Lavis JN, Hill SJ, Squires JE. Knowledge translation of research findings. Implement Sci. May 31 2012;7:50. 10.1186/1748-5908-7-50. 10.1186/1748-5908-7-50 PMC3462671 22651257

[31] Purnell TS et al. “Achieving Health Equity: Closing The Gaps In Health Care Disparities, Interventions, And Research,“ *Health Aff (Millwood)*, vol. 35, no. 8, pp. 1410-5, Aug 1 2016, 10.1377/hlthaff.2016.0158. 10.1377/hlthaff.2016.0158 27503965

[32] Brehaut JC, Eva KW. Building theories of knowledge translation interventions: use the entire menu of constructs. Implement Sci. Nov 22 2012;7:114. 10.1186/1748-5908-7-114. 10.1186/1748-5908-7-114 PMC3520870 23173596

[33] Eccles M, Grimshaw J, Walker A, Johnston M, Pitts N. “Changing the behavior of healthcare professionals: the use of theory in promoting the uptake of research findings,“ *J Clin Epidemiol*, vol. 58, no. 2, pp. 107 – 12, Feb 2005, 10.1016/j.jclinepi.2004.09.002. 10.1016/j.jclinepi.2004.09.002 15680740

[34] Etherington C, et al. Applying an intersectionality lens to the theoretical domains framework: a tool for thinking about how intersecting social identities and structures of power influence behaviour. BMC Med Res Methodol. Jun 26 2020;20(1):169. 10.1186/s12874-020-01056-1. 10.1186/s12874-020-01056-1 PMC7318508 32590940

[35] Presseau J, et al. Selecting implementation models, theories, and frameworks in which to integrate intersectional approaches. BMC Med Res Methodol. Aug 4 2022;22(1):212. 10.1186/s12874-022-01682-x. 10.1186/s12874-022-01682-x PMC9351159 35927615

[36] Birken SA et al. “T-CaST: an implementation theory comparison and selection tool,“ *Implement Sci*, vol. 13, no. 1, p. 143, Nov 22 2018, 10.1186/s13012-018-0836-4. 10.1186/s13012-018-0836-4 PMC6251099 30466450

[37] Strifler L, et al. Scoping review identifies significant number of knowledge translation theories, models, and frameworks with limited use. J Clin Epidemiol. Aug 2018;100:92–102. 10.1016/j.jclinepi.2018.04.008. 10.1016/j.jclinepi.2018.04.008 29660481

[38] Damschroder LJ, Aron DC, Keith RE, Kirsh SR, Alexander JA, Lowery JC. Fostering implementation of health services research findings into practice: a consolidated framework for advancing implementation science. Implement Sci. Aug 7 2009;4:50. 10.1186/1748-5908-4-50. 10.1186/1748-5908-4-50 PMC2736161 19664226

[39] Safaeinili N, Brown-Johnson C, Shaw JG, Mahoney M, Winget M (2020). CFIR simplified: pragmatic application of and adaptations to the Consolidated Framework for Implementation Research (CFIR) for evaluation of a patient-centered care transformation within a learning health system. Learn Health Syst.

[40] Abrams JA, Tabaac A, Jung S, Else-Quest NM. Considerations for employing intersectionality in qualitative health research. Soc Sci Med. Aug 2020;258:113138. 10.1016/j.socscimed.2020.113138. 10.1016/j.socscimed.2020.113138 PMC7363589 32574889

[41] Bauer GR. “Incorporating intersectionality theory into population health research methodology: challenges and the potential to advance health equity,“ *Soc Sci Med*, vol. 110, pp. 10 – 7, Jun 2014, 10.1016/j.socscimed.2014.03.022. 10.1016/j.socscimed.2014.03.022 24704889

[42] Bowleg L, Brooks K, Ritz SF (2008). Bringing home more than a paycheck: an exploratory analysis of black lesbians’ experiences of stress and coping in the workplace. J Lesbian Stud.

[43] Bowleg L, Huang J, Brooks K, Black A, Burkholder G (2003). Triple jeopardy and beyond: multiple minority stress and resilience among black lesbians. J Lesbian Stud.

[44] Bowleg L. “When Black + lesbian + woman ≠ Black lesbian woman: The methodological challenges of qualitative and quantitative intersectionality research,“ *Sex Roles*, vol. 59, no. 5–6, pp. 312 – 25, 2008.

[45] Vahabi M, Lofters A. Muslim immigrant women’s views on cervical cancer screening and HPV self-sampling in Ontario, Canada. BMC Public Health. Aug 24 2016;16(1):868. 10.1186/s12889-016-3564-1. 10.1186/s12889-016-3564-1 PMC4997745 27557928

[46] Board on Population Health and Public Health Practice Institute of Medicine., “Roundtable on the Promotion of Health Equity and the Elimination of Health Disparities: Leveraging Culture to Address Health Inequalities,“ 2013. [Online]. Available: https://www.ncbi.nlm.nih.gov/books/NBK201298/.

[47] Shelton RC, Adsul P, Oh A, Moise N, Griffith DM. “Application of an antiracism lens in the field of implementation science (IS): Recommendations for reframing implementation research with a focus on justice and racial equity. Implementation Research and Practice, 2,“ 2021. [Online]. Available: 10.1177/26334895211049482. 10.1177/26334895211049482 PMC9978668 37089985

[48] Curtis E, et al. Why cultural safety rather than cultural competency is required to achieve health equity: a literature review and recommended definition. Int J Equity Health. Nov 14 2019;18(1):174. 10.1186/s12939-019-1082-3. 10.1186/s12939-019-1082-3 PMC6857221 31727076

[49] Perez Jolles M, Willging CE, Stadnick NA et al. Understanding implementation research collaborations from a co-creation lens: Recommendations for a path forward. Front Health Serv. 2022;2:942658. 10.3389/frhs.2022.942658 PMC10003830 36908715

