# Supplementary material for: Developing the intersectionality supplemented Consolidated Framework for Implementation Research (CFIR) and tools for intersectionality considerations
Source: BMC Med Res Methodol. 2023 Nov 9;23:262. doi: 10.1186/s12874-023-02083-4 (PMC10636989; doi:10.1186/s12874-023-02083-4)
Supplement: Supplementary file 1 — Supplementary Material 1 [file 12874_2023_2083_MOESM1_ESM.docx]

| **File** | **Title** | **Description** |
| --- | --- | --- |
| **Supplementary File 1: Appendix A** | Overview of the prioritized Consolidated Framework for Implementation Research (CFIR) domains and constructs | Figure outlining the domains and constructs of the CFIR that were prioritized to intersectionality considerations. |

**Supplementary File 1:**

**Appendix A:** Overview of the prioritized Consolidated Framework for Implementation Research (CFIR) domains and constructs

**Intervention Characteristics**

- Intervention Source
- *Evidence Strength & Quality*
- **Relative Advantage**
- **Adaptability**
- *Trialability*
- **Complexity**
- *Design Quality & Packaging*
- **Cost**

**Outer Setting**

**Inner Setting**

**Characteristics of Individuals**

**Process of implementation**

- Patient Needs & Resources
- *Cosmopolitanism*
- Peer Pressure
- *External Policy & Incentives*
- **Structural Characteristics**
- Networks & Communications
- Culture
- Implementation Climate
  - **Tension for change**
  - Compatibility
  - Relative Priority
  - **Organizational Incentives & Rewards**
  - *Goals & Feedback*
- Readiness for implementation
  - **Leadership Engagement**
  - **Available Resources**
  - Access to Knowledge & Information

- *Knowledge & Beliefs about the Intervention*
- *Self-Efficacy*
- **Individual Stage of Change**
- Individual Identification with Organization
- Other Personal Attributes
- *Planning*
- **Engaging**
  - Opinion Leaders
  - Formally Appointed Internal Implementation Leaders
  - **Champions**
  - External Change Agents
- Executing
- *Reflecting & Evaluating*
