# Supplementary material for: Developing the intersectionality supplemented Consolidated Framework for Implementation Research (CFIR) and tools for intersectionality considerations
Source: BMC Med Res Methodol. 2023 Nov 9;23:262. doi: 10.1186/s12874-023-02083-4 (PMC10636989; doi:10.1186/s12874-023-02083-4)
Supplement: Supplementary file 2 — Supplementary Material 2 [file 12874_2023_2083_MOESM2_ESM.docx]

| **File** | **Title** | **Description** |
| --- | --- | --- |
| **Supplementary File 2** | CFIR Subgroup Intersecting Categories Survey Results | Results for a survey about intersecting categories. Respondents were members of the CFIR subgroup committee that developed the intersectionality supplemented CFIR. |

**Supplementary File 2:** CFIR Subgroup Intersecting Categories Survey Results

|  | **n** | **%** |
| --- | --- | --- |
| **Racial/ethnic identity** |  |  |
| White / Caucasian | 3 | 60% |
| Other | 2 | 40% |
| **Sex Assigned at Birth** |  |  |
| Female | 4 | 80% |
| Male | 1 | 20% |
| **Current Gender identity** |  |  |
| Female | 4 | 80% |
| Male | 1 | 20% |
| Other | 0 | 0% |
| **Sexual orientation** |  |  |
| Heterosexual | 4 | 80% |
| Queer (does not follow common sexual orientations) | 1 | 20% |
| Gay | 0 | 0% |
| Lesbian | 0 | 0% |
| Bisexual | 0 | 0% |
| Two-spirit | 0 | 0% |
| **Family Income** |  |  |
| Less than $60,000 | 0 | 0% |
| $60,000 - $89,999 | 0 | 0% |
| $90,000 - $119,999 | 1 | 20% |
| $120,000 - $149,999 | 1 | 20% |
| $150,000 or more | 3 | 60% |
| **Type of Housing** |  |  |
| Own home | 4 | 80% |
| Renting home | 1 | 20% |
| Other | 0 | 0% |
| **Place of residence** |  |  |
| Rural area, with a population less than 1,000 | 0 | 0% |
| Small population center, with a population between 1,000 and 29,999 | 1 | 20% |
| Medium population center, with a population between 30,000 and 99,999 | 0 | 0% |
| Large urban population center, with a population of 100,000 or more | 4 | 80% |
| **Education** |  |  |
| 12th grade or less | 0 | 0% |
| Graduated high school or equivalent | 0 | 0% |
| Completed college/university | 0 | 0% |
| Completed master’s degree | 2 | 40% |
| Completed doctorate | 3 | 60% |
| **Employment Status** |  |  |
| Full time employed | 5 | 100% |
| Part-time employed | 0 | 0% |
| Student | 0 | 0% |
| Unemployed (temporarily laid off, not employed for pay, etc.…) | 0 | 0% |
| **Marital status** |  |  |
| Married | 5 | 100% |
| In a civil partnership | 0 | 0% |
| Separated | 0 | 0% |
| Divorced | 0 | 0% |
| Widowed | 0 | 0% |
